# Epigenetic mechanisms and steroid-resistant nephrotic syndrome: The future potential for SRNS diagnosis

**DOI:** 10.1016/j.isci.2026.114973

**Published:** 2026-02-10

**Authors:** Yiying Zhu, Yinfeng Wang, Cuello Garcia Haider, Changling Cao, Ouzaouit Abdelhak, Yutong Fu, Huiqiang Huang, Tingya Jiang, Yang Zhou, Xiaoge Zhang, Yu Wu

**Affiliations:** 1 School of Life Sciences, Jiangsu University, Zhenjiang, China; 2 Nephrology Department, The Affiliated Xuzhou Municipal Hospital of Xuzhou Medical University, Xuzhou 221002, China; 3 AlloDx (Shanghai) Biotech Co., Ltd., Shanghai, China; 4 Department of Acupuncture and Moxibustion Rehabilitation, Danyang Hospital of Traditional Chinese Medicine, Zhenjiang 212300, China

**Keywords:** Nephrology, Molecular biology, Epigenetics

## Abstract

Nephrotic syndrome (NS) is commonly managed with glucocorticoid (GC) therapy, yet about 10%–30% of children do not achieve remission after an adequate initial steroid course and are classified as steroid-resistant nephrotic syndrome (SRNS); in adults, proportions are generally higher and heterogeneous across histologies. Currently, genetic testing can identify causative mutations in 30% of SRNS cases, highlighting the need for complementary pre-treatment stratification approaches. This review synthesizes human evidence linking epigenetic dysregulation to GC responsiveness, highlighting differential DNA methylation patterns in genes such as *NLRP3* and *SOCS3*. Simultaneously, the expression levels of microRNAs (miRNAs) such as miR-142 and miR-30 have been shown to be associated with the efficacy of GC treatment. We propose a multi-biomarker integrative analysis strategy that combines methylation profiles with miRNA expression and emerging histone modification signals for pre-treatment risk stratification and prediction of therapeutic response, thereby reducing ineffective steroid exposure and enabling mechanism-informed management pending prospective validation.

## Graphical abstract

**Figure:**
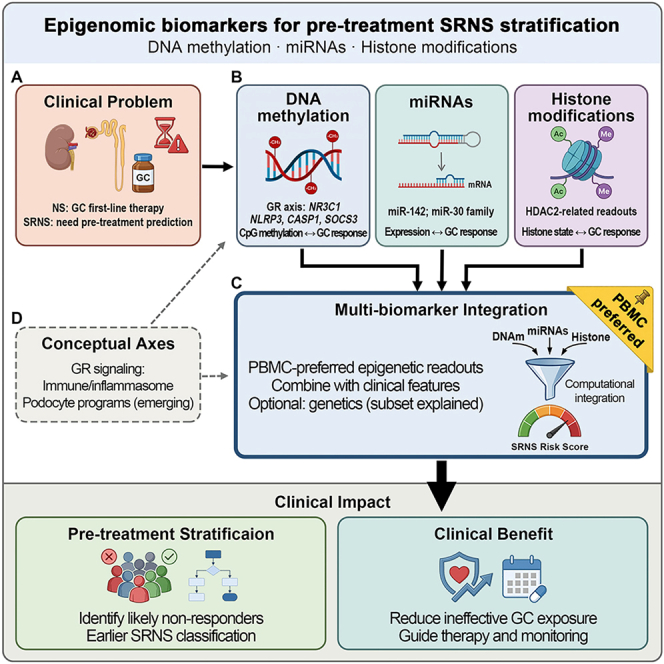

## Introduction

Nephrotic syndrome (NS) is a common pediatric kidney disorder with a global incidence of approximately 2 to 4.1 cases per 100,000 children.^1^ Across general populations, the typical incidence is around 2–7 per 100,000 child-years. Reported extremes span from 1.15 to 16.9 cases per 100,000 child-years, and the upper bound reflects specific ethnic subgroups/regions rather than most populations.^2^^,^^3^^,^^4^ NS is characterized by increased glomerular basement membrane permeability, presenting with massive proteinuria (>3.52 g/24 h), hypoalbuminemia (serum albumin <3.5 g/dL), and edema.^2^^,^^5^ Although glucocorticoid (GC) is the first-line treatment, about 10%–30% of children fail to remit after an adequate initial steroid course and are classified as steroid-resistant nephrotic syndrome (SRNS),^6^^,^^7^^,^^8^^,^^9^^,^^10^ with estimates differing across cohorts and diagnostic pathways; in adults, rates are generally higher by approximately 30%–40% and heterogeneous across histologies.^11^ In SRNS, approximately 30%–50% of patients progress to end-stage kidney disease (ESKD), increasing transplantation needs and mortality risk.^8^^,^^12^^,^^13^

Despite advances, early and accurate separation of steroid-sensitive nephrotic syndrome (SSNS) from SRNS remains challenging. Current diagnostic, which requires 4 weeks of adequate prednisone treatment in children and up to 16 weeks in adults, and therapeutic strategies often rely on empirical steroid treatment, which may delay effective management in patients with SRNS.^7^^,^^14^^,^^15^^,^^16^ This delay not only exposes patients to unnecessary side effects but also contributes to progressive renal damage.^17^ Kidney biopsy is not a uniform baseline test; its indication and timing vary across centers, and in most cases, it is performed only after SRNS has been diagnosed.^18^^,^^19^^,^^20^ As such, there is a growing need for more precise diagnostic tools and targeted therapies to improve outcomes and reduce the burden of ineffective interventions.

**Figure 1:**
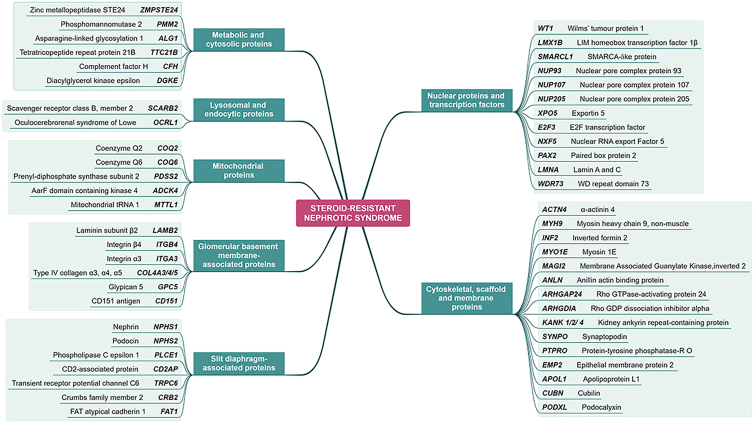
Representative mutated genes associated with SRNS categorized by functional protein groups This figure summarizes reported genetic mutations associated with SRNS, classified according to the functional types of encoded proteins, including metabolic and cytosolic proteins, lysosomal and endocytic proteins, mitochondrial proteins, glomerular basement membrane-associated proteins, slit diaphragm-associated proteins, cytoskeletal/membrane/scaffold proteins, and nuclear proteins and transcription factors. These mutations primarily affect podocyte integrity, signal transduction, and transcriptional regulation, reflecting the genetic heterogeneity and multifactorial nature of SRNS pathogenesis.

Multiple groups are evaluating early biomarkers to distinguish SRNS from SSNS and to reduce ineffective steroid exposure.^21^ With advences in next-generation sequencing (NGS), over 50 monogenic causes of NS/SRNS genes have been identified, most of which are podocyte expressed.^7^^,^^14^^,^^22^^,^^23^^,^^24^^,^^25^ This review summarizes the representative SRNS-related genes reported thus far, along with their functional classifications, to provide insights into the genetic basis and heterogeneity of the disease (Figure 1). In childhood-onset SRNS, NGS explains approximately 30% of cases; however, genetic yields are lower in adults and in certain populations without known monogenic risk.^26^^,^^27^^,^^28^^,^^29^^,^^30^^,^^31^ Likewise, genetic testing cannot account for epigenetic abnormalities arising from non-coding variants or environmental influences. At present, evidence is insufficient to establish epigenetic alterations as the primary cause of the approximately 70% of SRNS cases with unknown etiology. These cases may also involve undetected monogenic variants, polygenic inheritance, or unmeasured environmental exposures.

Case reports demonstrate robust steroid responses in some NS phenotypes such as IgA nephropathy^32^^,^^33^ and podocyte unfolding glomerulopathy.^34^ Nonetheless, a considerable proportion of patients exhibit steroid resistance, and prolonged exposure to ineffective steroids can cause toxicity and may result in irreversible kidney injury. Adverse event rates increase with prolonged exposure, underscoring the need to minimize ineffective steroid use.^35^

To address this issue, clinicians have explored alternative therapeutic strategies, including investigational integration of traditional Chinese medicine (TCM) with Western therapies. Li et al. proposed that TCM may exert therapeutic effects in NS through immunomodulatory and renoprotective mechanisms, and the approach remains in investigation and requires further validation.^36^^,^^37^ Additionally, a meta-analysis of 18 randomized controlled trials indicated that tacrolimus and cyclosporine may be optimal first-line immunosuppressants for children with SRNS, although larger studies are needed to confirm their efficacy and safety profiles.^38^ While tacrolimus and ciclosporin are first-line for SRNS, regional variation exists in clinical practice and in the evidence base, and these drugs have strong but not universal efficacy with approximately 10%–20% of patients being multidrug resistant—the population at most risk of chronic kidney disease (CKD) and ESKD.^38^^,^^39^

Despite use, current options remain suboptimal, with limited efficacy—remission rates of only approximately 40%–60%—and are often associated with significant adverse effects, including increased risks of infection, nephrotoxicity, and metabolic disturbances.^40^ Furthermore, 30%–50% of SRNS patients relapse following kidney transplantation, further underscoring the inadequacy of current treatment options.^12^^,^^13^^,^^40^ These gaps motivate mechanistic studies and development of pre-treatment diagnostic biomarkers.

This review synthesizes human evidence on the epigenetic regulation of steroid response in SRNS and evaluates its translational potential for pre-treatment diagnostics. We distinguish established clinical practice from investigational approaches and outline a framework that integrates genetics with epigenomics to guide earlier mechanism-based therapy and limit ineffective steroid exposure.

## Epigenomics: A novel perspective for SRNS diagnostic research

**Figure 2:**
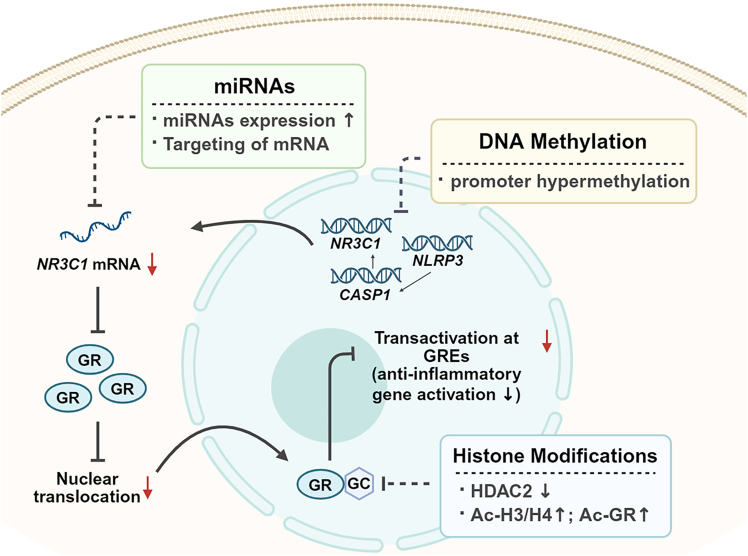
Integrated epigenetic alterations impacting the glucocorticoid receptor pathway in SRNS Upregulated miRNAs target *NR3C1* mRNA, reducing GR abundance and nuclear translocation. DNA methylation changes (illustrated as promoter hypermethylation at loci such as *NR3C1*, *NLRP3*, and *CASP1*) together with histone changes (decreased HDAC2, increased Ac-H3/H4 and Ac-GR) attenuate GR-mediated transactivation at glucocorticoid response elements (GREs), lowering anti-inflammatory gene activation.

Epigenomics provides a complementary perspective for SRNS diagnostics by capturing regulatory changes not explained by coding variants, and such alterations may contribute to disease onset and progression and may modulate steroid response (Figure 2). Existing studies suggest that DNA methylation may attenuate GC responsiveness by modulating genes within the glucocorticoid receptor (GR) pathway (*NR3C1*) or within the inflammasome pathway such as *NLRP3* and *CASP1*.^41^^,^^42^ MicroRNA (miRNA) may regulate GR expression by promoting *NLRP3* mRNA degradation or translational repression and by modulating the expression of podocyte-associated genes and proteins, thereby potentially influencing steroid responsiveness.^43^ In parallel, post-translational histone modifications offer novel molecular insights into the mechanisms underlying SRNS. The dynamic and potentially reversible nature of epigenetic marks highlights opportunities for pre-treatment risk stratification, early diagnosis, and therapy monitoring.^44^

In current biomedical research, multi-omics biomarker integration strategies are increasingly employed, combining two or more omics layers—including genomics, transcriptomics, and proteomics—as well as epigenomics approaches such as DNA methylation, miRNA expression, and histone modifications. These strategies aim to identify robust biomarkers for disease diagnosis, prognosis, and treatment. For instance, in a study of acute lymphoblastic leukemia (ALL), models that integrate genomics, transcriptomics, DNA methylation, and miRNA data in final multivariate analysis improved the discrimination of GC-sensitive versus resistant cases and enhanced subtype classification and risk prediction, providing a methodological precedent.^45^ Guided by these precedents, we outline an SRNS framework that integrates methylation profiles, miRNA expression, and histone modification data with clinical features to enable early identification of non-responders. This review synthesizes human evidence on epigenomic changes in SRNS and evaluates their translational potential for early diagnostics, while distinguishing established clinical practice from research-stage approaches and proposing a pre-treatment risk-stratification framework.

## DNA methylation: A key epigenetic regulatory factor in SRNS

### Mechanisms of DNA methylation in regulating gene expression

In mammals, DNA methylation is a key epigenetic regulatory mechanism,^46^ primarily catalyzed by DNA methyltransferases (DNMTs), which add a methyl group at the 5-position of cytosine to form 5-methylcytosine (5-mC).^47^ Although 5-mC constitutes only about 1% of nucleotides in the human genome, most DNA methylation occurs at cytosine residues preceding guanine (CpG dinucleotides).^47^ DNMT activity may modulate gene transcription, particularly within promoter regions, with effects that are context dependent.^48^ Genomic regions with a high density of CpG sites, termed CpG islands and first defined by Gardiner-Garden and Frommer, are commonly located within or near vertebrate gene promoter.^49^^,^^50^ DNA methylation regulates gene expression through multiple mechanisms, including (1) altering the conformation of the DNA molecule, thereby affecting transcription factor binding^51^; (2) recruiting methyl-CpG-binding domain proteins, such as MeCP2, which modulate chromatin structure and gene transcription^52^^,^^53^; and (3) blocking protein-DNA interactions at regulatory elements.^54^ These mechanisms collectively regulate gene activity, influencing diverse cellular functions and contributing to the development and progression of various diseases.^55^

### Aberrant DNA methylation patterns of SRNS-associated genes

The role of DNA methylation has been extensively investigated in various diseases, particularly in the fields of oncology, autoimmune disorders, and metabolic diseases.^56^ In recent years, a growing body of research has begun to explore its potential involvement in SRNS, highlighting its emerging relevance in renal pathology. This article summarizes some reported studies on potential methylation biomarkers (Table 1).

**Table 1:** Methylation diagnostic modalities and biomarkers that have been reported

Disease	Sample	Biomarker	Method	Reference
Kidney cancer	urine	*TFAP2B*, *TAC1*, *PCDH8*, *ZNF677*, *FLRT2*, and *PCDH8*	methylation-specific PCR	Žalimas et al. and Li et al.^57^^,^^58^
Bladder cancer	urine	*PCDH17*, *POU4F2*, *PENK*, and *DMRTA2*	sequence Illumina, methylation-specific PCR	Fang et al. and Deng et al.^59^^,^^60^
Lung cancer	plasma	*SHOX2*	qPCR	Li et al.^61^
	tumor tissue	*TMEM196* and *L1RE1*	Illumina methylation assay	Walter et al. and Duan et al.^62^^,^^63^
Breast cancer	blood	*CTSZ*	MALDI-TOF mass, spectrometry	Li et al.^64^
Esophageal cancer	saliva	*CTNND2* and *CCL20*	scRNA-seq	Maity et al.^65^
Hepatic cancer	serum	*P16*, *RASSF1A*, *FHIT*, *TBX2*, and *ZNF154*	methylation-specific PCR	Cozma et al. and Bai et al.^66^^,^^67^
Alzheimer’s disease	blood	*CSF*	next-generation sequencing	Chang et al., Zhang et al., and Hu et al.^68^^,^^69^^,^^70^
Colorectal cancer	stool	*SEPT9*, *NDRG4*, and *BMP3*	methylation-specific PCR	Liu et al.^71^

*TFAP2B*, transcription factor AP-2 beta; *TAC1*, tachykinin precursor 1; *PCDH8*, protocadherin 8; *ZNF677*, zinc finger protein 677; *FLRT2*, fibronectin leucine-rich transmembrane protein 2; *PCDH17*, protocadherin 17; *POU4F2*, POU class 4 homeobox 2; *PENK*, proenkephalin; *DMRTA2*, DMRT like family A2; *SHOX2*, SHOX homeobox 2; *TMEM196*, transmembrane protein 196; *L1RE1*, LOC110251006 uncharacterized LOC110251006; *CTSZ*, cathepsin Z; *CTNND2*, catenin delta 2; *CCL20*, C-C motif chemokine ligand 20; *P16*, cyclin-dependent kinase inhibitor 2A; *RASSF1A*, Ras association domain family member 1; *FHIT*, fragile histidine triad diadenosine triphosphatase; *TBX2*, T-box transcription factor 2; *ZNF154*, zinc finger protein 154; *CSF*, colony-stimulating factor; *SEPT9*, septin 9; *NDRG4*, NDRG family member 4; *BMP3*, bone morphogenetic protein 3.

#### GR/*NR3C1* axis

*NR3C1* encodes the GR, placing this locus at the core of GC signaling in NS. Methylation changes around the GR pathway have been shown to be associated with differences in steroid responsiveness.

Likewise, aberrant methylation and expression of the GR-encoding gene *NR3C1* have been strongly linked to resistance to GC, as demonstrated in various cancers and immune-related diseases.^41^^,^^42^ Hypermethylation in specific promoter regions of *NR3C1* and *FKBP52* significantly suppresses gene transcription, resulting in reduced expression of GR and potentially contributing to steroid resistance.^72^

Mechanistically, methylation can alter local DNA structure and transcription factor occupancy, recruit methyl-CpG-binding proteins such as MeCP2 to remodel chromatin, and hinder protein-DNA interactions at regulatory elements; these processes can operate at the *NR3C1* promoter and near glucocorticoid response elements (GREs).^73^^,^^74^^,^^75^ In human studies, the observed links remain predominantly correlational, with effect sizes varying across platforms and cohorts, and causal relationships have not been established.^76^ Interpretation therefore requires attention to cell-type composition, the timing of sampling relative to GC exposure, and batch effects; standardized multi-cohort validation will be needed before *NR3C1*-adjacent methylation can inform pre-treatment risk stratification.^77^^,^^78^

#### Immune pathways: *SOCS*, *NLRP3*, and *CASP1*

In the immune axis relevant to steroid responsiveness, we focus on the inflammasome arm (NLRP3-CASP1) and the cytokine-feedback arm (SOCS-JAK/STAT).^79^^,^^80^ DNA methylation has been reported to be associated with altered activity of *NLRP3* and *CASP1*, providing a plausible link between epigenetic regulation and inflammatory tone that may shape steroid responsiveness.^81^

Multiple epigenetic studies have revealed that gene-specific DNA methylation-induced expression changes, along with non-coding RNAs and post-translational histone modifications, may be associated with autoinflammatory and inflammation-related diseases by providing a novel mechanism for controlling inflammasome activity.^82^ The suppression or activation mediated by DNA methylation of inflammasomes such as *NLRP1*, *NLRP3*, and *AIM2* plays a critical role in determining disease progression and severity.^82^^,^^83^ In juvenile spondyloarthritis, Lamot et al. used methylated DNA immunoprecipitation to assess methylation status and found that hypermethylation of the *NLRP3* promoter reduced its protein expression and contributed to gut microbiota dysbiosis.^84^ In kidney diseases, activation of the *NLRP3* inflammasome exacerbates podocyte injury and is implicated in the progression of diabetic nephropathy.^85^^,^^86^

In studies on GC resistance, Paugh et al. found that primary leukemic cells from 444 newly diagnosed ALL patients were sensitive to prednisolone, a response associated with hypomethylation of the *NLRP3* and *CASP1* promoters, which can alter gene expression. Increased expression of *CASP1* promotes GR cleavage, attenuating hormone-induced transcription and contributing to resistance, suggesting that this mechanism may also be present in other GC-resistant conditions.^79^ Based on this, subsequent research in 10 SRNS and 18 patients with SSNS revealed significant hypomethylation of the *NLRP3* promoter in SRNS, the site cg21991396 showing a strong predictive value for GC resistance, effectively distinguishing sensitive from resistant cases in pediatric and adult populations (receiver operating characteristic [ROC] ≥ 73.5%, *p* ≤ 0.00097).^17^

Within the cytokine-feedback arm, *SOCS1/SOCS3* act as classical negative regulators of JAK/STAT; promoter-proximal methylation has been shown to be associated with reduced *SOCS* expression, a state that is compatible with sustained STAT activation and pro-inflammatory signaling.^80^^,^^87^ Conceptually, methylation could influence priming and activation, thereby modulating the magnitude or persistence of inflammasome signaling.^88^^,^^89^ In human SRNS studies, most signals remain correlational, and estimates vary across matrices and platforms; interpretation requires attention to cell-type composition, timing of sampling relative to GC exposure, and batch effects.^90^^,^^91^ Taken together, *SOCS*- and inflammasome-related methylation provides a complementary mechanistic anchor to *NR3C1* for pre-treatment risk stratification, pending standardized, multi-cohort prospective validation.^77^

An early study by Nowicka et al. included a cohort of 20 patients with SRNS and 24 patients with SSNS and reported significantly increased expression of *SOCS3* and *SOCS5* mRNA in the SRNS group, suggesting their potential as predictive biomarkers for resistance to steroids.^92^ Subsequently, the same group evaluated the promoter methylation status of *SOCS3* and *SOCS5* in 40 SRNS and 36 SSNS patients and found that the methylation level of the *SOCS3* promoter in SRNS patients was significantly lower than in both the SSNS and healthy control groups (*p* < 0.0001), suggesting that *SOCS3* promoter hypomethylation may serve as a biomarker for the early identification of SRNS.^90^ In a follow-up study published in 2021, the researchers enrolled 124 patients with NS comprising 53 cases of SRNS, 71 cases of SSNS, and 31 cases of steroid-dependent NS, including 75 patients from their earlier studies. They analyzed 11 SNPs, 6 mutations, and the *SOCS3* promoter methylation profile, finding that the SRNS group exhibited a 15-fold higher incidence of complete unmethylation in *SOCS3* methylation region 2 compared to controls (*p* < 0.0001), while no significant difference was observed in region 1.^93^

#### Podocyte injury

Podocyte homeostasis critically depends on the integrity of slit diaphragm and cytoskeletal/adhesive gene networks, particularly including *NPHS1* (nephrin), *NPHS2* (podocin), *CD2AP*, *ACTN4*, *TRPC6*, and *INF2*, alongside transcription chromatin regulators *WT1* and *MAFB*, which collectively govern podocyte-specific gene expression programs.^94^^,^^95^ While pathogenic coding variants in these genes account for subsets of familial and monogenic SRNS and focal segmental glomerular sclerosis (FSGS), the role of DNA methylation or other epigenetic modifications at these loci in SRNS remains understudied. Recent reviews by Kaori on podocyte DNA methylation highlight that methylation changes are associated with alterations in nephrin expression, cytoskeletal stability, and podocyte foot-process morphology.^96^

Emerging human data demonstrate that podocyte DNA damage and global glomerular DNA methylation changes correlate with proteinuria severity and eGFR decline.^97^ These observations support a model in which epigenetic states in podocytes regulate both the structural filtration barrier and the cellular environment for GC responsiveness. In particular, *MAFB-*driven chromatin opening allows *WT1* binding to the *NPHS1* and *NPHS2* promoters, suggesting that promoter or enhancer accessibility at slit-diaphragm loci may determine podocyte susceptibility to injury or therapeutic response.^98^

However, to date, methylation signatures at podocyte-related genes have not been robustly validated in human SRNS cohorts. Thus, we propose that future studies prioritize pre-treatment methylation profiling in podocyte-enriched sample types. These efforts will help assess whether epigenetic modulation of podocyte gene networks can be used for steroid resistance risk stratification and personalized therapy in SRNS.

**Figure 3:**
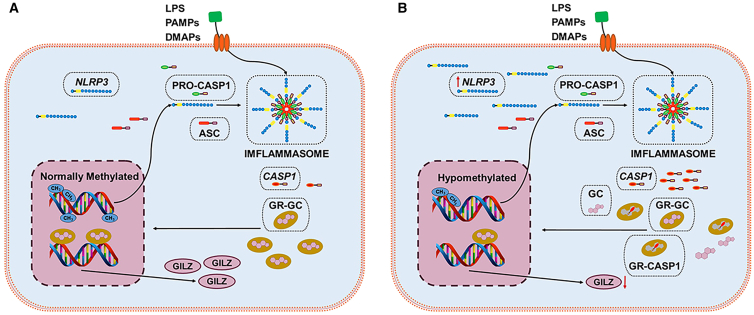
Molecular mechanism by which NLRP3 gene hypomethylation and CASP1-mediated cleavage of GR (NR3C1 coding) contribute to glucocorticoid resistance (A) Under normal methylation conditions, the promoter region of *NLRP3* is methylated, suppressing its transcriptional activity. As a result, the activation of pro-caspase-1 (pro-CASP1) is limited, leading to low expression levels of *CASP1*, and inflammatory cytokines IL-1β and IL-18 remain at physiological levels. GC can effectively bind to the GR, promoting GILZ expression and exerting anti-inflammatory effects. (B) In patients with SRNS, hypomethylation of the *NLRP3* promoter leads to its overexpression and subsequent activation of pro-CASP1, resulting in excessive *CASP1* production. High levels of *CASP1* inhibit the binding of GC to GR, disrupt GR signaling, and reduce GILZ expression, ultimately contributing to the development of GC resistance. LPS, lipopolysaccharide treatment; PAMPs, pathogen-associated molecular patterns; DAMPs, molecular patterns associated with damage; *NLRP3*, NLR family pyrin domain containing 3; ASC, inflammatory-associated signal transduction protein; ASC, apoptosis-associated speck-like protein containing a caspase recruitment domain (CARD); *CASP1*, caspase-1, capable of cutting glucocorticoid receptors; GC, glucocorticoid; GR, glucocorticoid receptor; GILZ, glucocorticoid-induced leucine zipper.

These findings highlight a potential regulatory interplay between *NLRP3*, *CASP1*, and *NR3C1* at both the methylation and expression levels (Figure 3).^17^^,^^79^ Together, methylation-driven regulation of *SOCS3*, *NLRP3*, *CASP1* and *NR3C1* may play a pivotal role in the development of GC resistance in kidney diseases.

### Role of DNA methylation in peripheral blood mononuclear cells in disease subtyping

**Figure 4:**
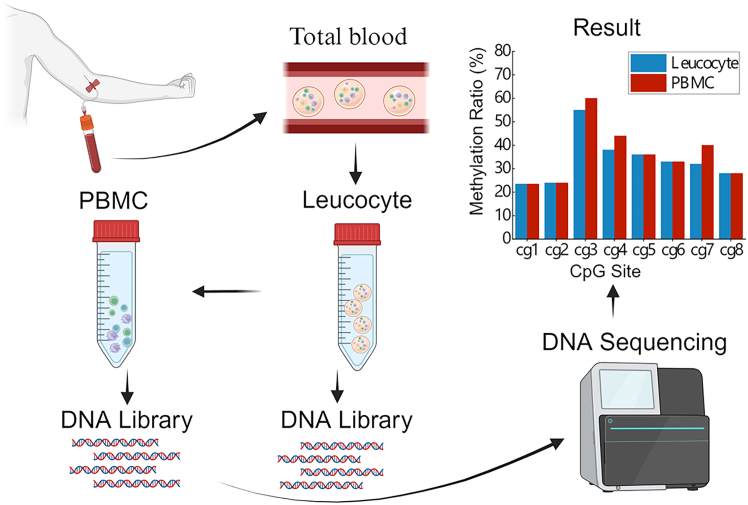
DNA extracted from whole blood and PBMC exhibited different levels of methylation at specific CpG sites after the creation of DNA libraries and methylation sequencing

Current DNA methylation studies predominantly rely on whole blood samples; however, the cellular heterogeneity inherent to whole blood may mask cell-type-specific methylation signatures and necessitate considerable financial and labor resources. Given the disease lesions, a targeted methylation analysis of specific tissues or cell types may offer greater precision. Peripheral blood mononuclear cells (PBMCs), comprising lymphocytes and monocytes,^99^ represent a heterogeneous cell population^100^ and exhibit distinct methylation patterns compared to whole blood, suggesting that PBMCs should be prioritized in methylation studies of inflammatory diseases.^101^ In a study by Yosra et al., DNA methylation profiles were analyzed in Down syndrome patients with and without breast cancer, focusing on CpG sites in T21-BC and T21-BCF groups. A total of 32,973 significant CpG sites were identified in whole blood DNA and 26,403 in PBMCs, with only 3,993 sites overlapping between the two types of sample.^102^ For example, methylation levels at specific CpG sites were significantly different between whole blood and PBMC-derived DNA.^102^^,^^103^ These findings indicate that PBMCs may offer superior accuracy for methylation analysis. The heterogeneity in the methylation patterns between whole blood and PBMC DNA can substantially impact analytical outcomes (Figure 4).

The onset of NS is strongly associated with immune dysregulation. In studies on SRNS, the proportion of CD19^+^ B lymphocytes has been shown to increase significantly during the active phase of resistance to steroids and decrease during remission.^104^ Given the crucial role of the immune system in the pathogenesis of NS,^105^^,^^106^^,^^107^^,^^108^ DNA methylation studies targeting lymphocytes may offer a greater value in elucidating the underlying mechanisms of SRNS.

Currently, most studies on GC resistance focus on DNA methylation in whole blood,^109^ while PBMC-specific methylation data for SRNS-related genes remain scarce.^110^ Given the higher cellular specificity of PBMCs, future methylation analyses in SRNS should prioritize PBMC-derived DNA, as it is more likely to capture patient-specific methylation signatures accurately.

## miRNAs: Post-transcriptional regulators in SRNS

### Biogenesis of miRNAs and their epigenetic regulatory functions

miRNA-mediated gene silencing is recognized as a post-transcriptional arm of epigenetic regulation.^111^ These molecules are small, non-coding single-stranded RNAs, typically 19–25 nucleotides long, that are broadly expressed in eukaryotic cells and regulate gene expression at multiple levels.^112^ Functionally, miRNAs bind to complementary sites within the 3′ untranslated region (3′ UTR) of target mRNAs, causing mRNA decay or translational repression and thereby enabling fine-tuned post-transcriptional control of gene expression.^113^^,^^114^ Beyond sequence-based targeting, miRNA activity is dynamically modulated by epitranscriptomic marks such as N^6^-methyladenosine (m^6^A) methylation, which can influence miRNA stability and function. Their biogenesis is controlled by RNase III enzymes Drosha and Dicer, forming an integrated, highly coordinated pathway from transcription to function.^115^ Importantly, miRNAs are detectable not only intracellularly but also as stable species in diverse extracellular fluids, such as plasma, serum,^116^^,^^117^ saliva,^118^ urine,^119^^,^^120^ and amniotic fluid,^121^ highlighting their potential as non-invasive diagnostic and monitoring biomarkers.

### Diagnostic and predictive miRNA biomarkers in SRNS

In addition to DNA methylation, miRNAs hold significant promise as diagnostic and prognostic biomarkers, making them powerful tools for disease detection and treatment.^122^ Over the past decade, quantitative and qualitative analyses of miRNA expression profiles have become a major focus in biomedical research, as aberrant miRNA expression is implicated in cancers, cardiovascular diseases, and immune disorders—informing biomarker discovery and the development targeted therapies.^123^ Because of their low molecular complexity, tissue-specific expression, and high stability, miRNAs serve as informative indicators of diverse physiological and pathological states.^124^

Previous studies have either concentrated on a limited number of specific miRNAs or used high-throughput approaches, such as microarrays and RNA sequencing (RNA-seq), to profile thousands of miRNAs.^125^^,^^126^^,^^127^^,^^128^ Here, we synthesize several reported studies investigating potential miRNA biomarkers (Table 2).

**Table 2:** MicroRNA diagnostic modalities and biomarkers that have been reported

Disease	Sample	Biomarker	Method	Reference
Asthma	serum	miR-155-5p, miR-532-5p	small RNA sequencing	Li et al.^129^
Colorectal cancer (CRC)	tissues and stool	miR-21, miR-92a	–	Wu et al.^130^
Heart transplant rejection	serum	miR-10a, miR-31	RT-qPCR	Duong Van Huyen et al.^131^
Cardiac arrest	serum and plasma	miR-124-3p	short RNA sequencing and PCR	Devaux et al.^132^
Papillary thyroid carcinoma (PTC)	serum	miR-146a-5p, miR-221-3p	digital PCR	Verrienti et al.^133^
Hepatitis C virus (HCV)	serum	miR-27A, miR-18b	RT-qPCR	Rashad et al.^134^
Hemodialysis (HD)	serum	miR-122-5p	RT-qPCR	Duni et al.^135^
Esophageal squamous cell carcinoma (ESCC)	salivary	miR-4505, miR-142-3p	microarray	Li et al.^136^

The aforementioned studies indicate that specific miRNAs, such as miR-125b, miR-186, and miR-142-5p, exhibit significant differential expression between SSNS and SRNS patients. In a prospective study, Liu et al. collected plasma samples from 51 patients with FSGS before and after corticosteroid therapy. Initial screening using the TaqMan low-density array platform assayed 384 miRNAs in plasma from 9 patients with FSGS and 9 healthy controls, revealing several miRNAs significantly upregulated in FSGS. Subsequent validation by quantitative reverse-transcription polymerase chain reaction (RT-qPCR) with TaqMan probes identified miR-125b, miR-186, and miR-193a-3p as elevated in these patients. Notably, in SSNS patients achieving complete remission after corticosteroid therapy, miR-125b and miR-186 levels decreased significantly (*p* = 0.002), while no significant changes were observed in patients with SRNS.^137^ In 2021, Bayomy et al. investigated the expression of five miRNAs—miR-142-5p, miR-191a-5p, miR-181-5p, miR-30a-5p, and miR-150a-5p—in peripheral blood from 80 pediatric NS patients and 100 healthy controls using RT-qPCR. All five miRNAs were significantly upregulated in patients with NS compared with controls. Consistently, a clear differential expression was observed between the SRNS and SSNS subgroups. Among these, miR-142-5p demonstrated the highest diagnostic precision, with an area under the curve (AUC) of 0.965 to distinguish NS patients from controls and an AUC of 1.00 for differentiating SRNS from SSNS. The panel comprising miR-142-5p, miR-181-5p, and miR-30a-5p was identified as the most effective for both initial NS diagnosis and prediction of steroid response, providing a promising basis for future SRNS diagnostics.^138^

In parallel, miRNAs can regulate the GC response by targeting GR (*NR3C1*) expression.^43^ Wang et al. demonstrated in a depression study that miR-124-3p directly binds the 3′ UTR of *NR3C1* transcripts in HEK293 cells, thus reducing GR expression and modulating the transcription of *NR3C1*.^139^ Similarly, Simone Kreth and colleagues revealed that miR-124 suppresses GRα expression, diminishing the anti-inflammatory effects of GCs and creating a feedback loop whereby GCs upregulate miR-124, which further exacerbates corticosteroid resistance in sepsis. Compared with SSNS patients, SRNS patients exhibit downregulated GRα expression in podocytes.^140^ In acute T cell lymphoblastic leukemia (T-ALL), Huang et al. found that miR-142-3p targets the 3′ UTR of GRα mRNA, inhibiting its translation and reducing protein levels, thereby inducing GC resistance; importantly, exogenous introduction of a miR-142-3p inhibitor restored GC sensitivity by reactivating GRα-mediated signaling pathways, suggesting a potential approach to reverse GC resistance.^141^ Separately, Wai-Yee Chan et al. reported that miR-192-3p negatively regulates GR by targeting *NR3C1* mRNA in fatty liver disease. This inhibition disrupts the GC-GR signaling pathway involved in lipogenesis; while the GC-GR axis promotes fatty liver through upregulation of lipogenic genes, miR-192-3p antagonizes GR expression and thus counteracts this process.^142^

**Figure 5:**
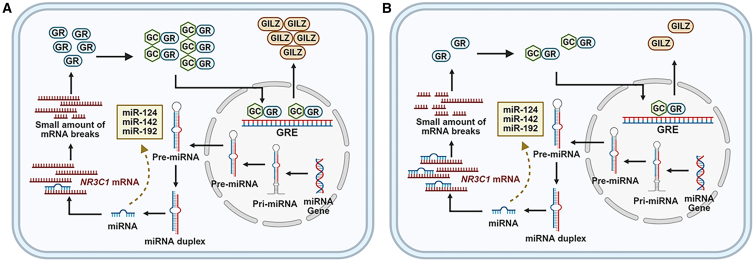
Suppression of GR signaling by miRNA overexpression in SRNS (A) Under normal conditions, GC bind to the GR, which translocates into the nucleus and activates anti-inflammatory gene expression, contributing to effective steroid responsiveness. (B) In SRNS, elevated levels of specific miRNAs (e.g., miR-124, miR-142, and miR-192) target *NR3C1* mRNA, leading to reduced GR expression and impaired nuclear translocation. This disruption in GR signaling attenuates downstream anti-inflammatory responses and contributes to steroid resistance. GC, glucocorticoid; GR, glucocorticoid receptor.

Importantly, certain miRNAs, including miR-124, miR-142-3p, and miR-192-3p, have been demonstrated to contribute to the molecular mechanisms of steroid resistance by targeting and downregulating the GC receptor GRα (Figure 5). Taken together, these findings highlight the potential of miRNAs as both predictive biomarkers and molecular classifiers in SRNS, while underscoring the need for further investigation and clinical validation.

## Histone modifications: Chromatin-level regulatory mechanisms in SRNS

Histones dynamically regulate gene expression through post-translational modifications such as acetylation, deacetylation, and methylation.^143^ As core structural components of chromatin, histones (H2A, H2B, H3, and H4) assemble with DNA to form nucleosomes.^144^ In addition to DNA methylation, histone acetylation and methylation are among the epigenetic marks studied most extensively. Histone acetylation in euchromatic regions, catalyzed by histone acetyltransferases, promotes transcriptional activation, while histone deacetylases (HDACs) remove these marks, leading to transcriptional repression and downregulation of pro-inflammatory genes commonly upregulated in disease contexts.^145^

This article summarizes several reported studies on potential histone biomarkers (Table 3). In nephrology-related research, Zhang et al. demonstrated that loss of histone H3 lysine 27 trimethylation (H3K27me3) in adult glomeruli promotes post-mitotic podocyte dedifferentiation and accelerates glomerular disease progression, while increased levels of H3K27me3 exert protective effects. These findings highlight the critical role of histone modification alterations in the development of glomerular disease, suggesting that inhibition of lysine-specific demethylases may beneficially modulate histone patterns and potentially slow disease progression.^146^

**Table 3:** Histone diagnostic modalities and biomarkers that have been reported

Disease	Sample	Biomarker	Method	Reference
Septic shock	PBMC	H3K18	RT-qPCR	Chu et al.^147^
Triple-negative breast cancer	tissues	H4K12	immunoblotting and immunohistochemistry	Cui et al.^148^
Gastric adenocarcinoma	tissues	H3K9	immunohistochemistry	Park et al.^149^
Pancreatic cancer	tissues	H3K18	western blotting	Hou et al.^150^
Glioma	tissues	H3K36me2	immunohistochemistry	Cong et al.^151^
Septic liver damage	tissues	H3Cit	immunohistochemistry	Nomura et al.^152^
Malignant peripheral nerve sheath tumor	tissues	H3K27	immunohistochemistry	Marchione et al.^153^
Breast cancer	cell	H4K20me3	immunohistochemistry	Yokoyama et al.^154^

H3K18, histone H3 lysine 18; H4K12, histone H4 lysine 12; H3K9, histone H3 lysine 9; H3K36me2, dimethylation of histone H3 lysine 36; H3Cit, citrullinated histone H3; H3K27me3, trimethylation of histone H3 lysine 27; H4K20me3, histone H4 lysine 20 trimethylation.

Abnormal histone modifications may contribute to the pathogenesis of SRNS through epigenetic regulation. Guan et al. examined the relationship between histone deacetylase 2 (HDAC2) expression and GC resistance by measuring HDAC2 expression and enzymatic activity in peripheral blood lymphocytes from 48 newly diagnosed pediatric primary NS patients including 25 SSNS and 23 SRNS cases—prior to GC treatment. Results showed that both HDAC2 expression and activity were significantly lower in the SRNS group than in the SSNS and healthy control groups pre- and post-therapy using GC (*p* < 0.01), while in the SSNS group, both parameters increased significantly after treatment compared to baseline (*p* < 0.01). These findings suggest that GC responsiveness is influenced by HDAC2 expression and enzymatic activity, indicating that HDAC2 may serve as a potential biomarker to predict steroid responsiveness in pediatric NS.^155^

In studies related to GC resistance, increasing acetylation of histone H3 and H4 has been shown to be associated with higher expression of genes that attenuate GC efficacy, thereby contributing to GC resistance. Yu et al. reported that the expression levels of the acetylated GR (Ac-GR), histone 3 (Ac-H3), and histone 4 (Ac-H4) differed significantly between healthy controls, patients with SSNS, and SRNS patients. In a cohort of 32 pediatric patients with SSNS and 15 pediatric patients with SRNS, baseline expression levels of these markers were significantly different (*p* < 0.05). Following GC therapy, SSNS patients exhibited marked reductions in Ac-GR (mean = 0.429, SD = 0.107, *p* = 8.41E−6), Ac-H3 (mean = 0.652, SD = 0.126, *p* = 5.38E−8), and Ac-H4 (mean = 0.599, SD = 0.098, *p* = 1.24E−7), while SRNS patients showed significant post-treatment increases, particularly in Ac-GR (mean = 0.498, SD = 0.113, *p* = 8.81E−3).^156^

**Figure 6:**
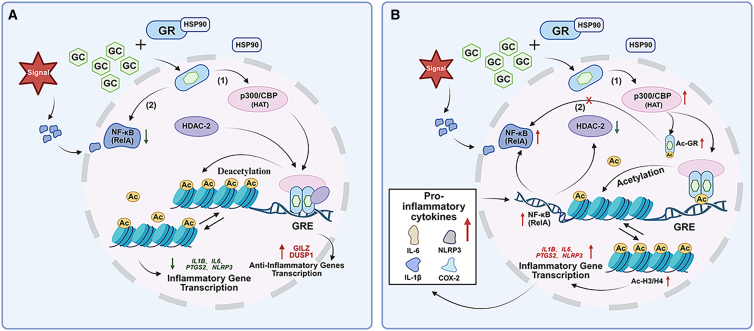
Histone acetylation balance and glucocorticoid response (A) GC-sensitive state: the GC-GR complex translocates to the nucleus; HDAC2 counterbalances p300/CBP, promoting deacetylation near GREs and enabling GR-mediated transcription of anti-inflammatory genes while restraining NF-қB (RelA)-driven inflammatory transcription. (B) GC-resistant state with acetylation imbalance: reduced HDAC2 and relatively increased p300/CBP activity raise Ac-GR and Ac-H3/H4, favoring NF-қB activation and transcription of pro-inflammatory genes, with diminished GR transactivation at GREs.

These findings highlight histone acetylation as a complementary regulatory axis alongside GR/*NR3C1* signaling for pre-treatment risk stratification, pending prospective validation (Figure 6). Overall, histone-based mechanisms and biomarkers in SRNS remain an emerging area with predominantly associative evidence from small cohorts; prospective, multi-center validation will be required before clinical translation.

## Summary and future perspectives

With the growing understanding of SRNS pathogenesis, epigenetic regulation has gained increasing attention as a key mechanism beyond genetic mutations, showing promising potential in early diagnosis and targeted therapy, while its clinical utility remains to be established through standardized, prospective multi-cohort validation. Although current studies are often limited by small sample sizes, cross-sectional or single-center designs, and a lack of external validation and assay standardization, the existing evidence provides a valuable foundation for future research. Moreover, interpretation is complicated by cell-type specificity, sampling-matrix effects, and heterogeneous platforms and analytic pipelines.

Among current studies, miR-142-5p has emerged as a repeatedly reported candidate biomarker capable of distinguishing SSNS from SRNS, while also reported to influence GR expression, yet it requires multi-cohort prospective validation before clinical use. In terms of DNA methylation, hypomethylation of the *SOCS3* and *NLRP3* promoter regions, as well as hypermethylation of the GR-encoding gene *NR3C1*, has been reported to associate with steroid resistance in observational studies, with effect sizes varying across matrices and platforms. Compared to whole blood DNA, PBMC offers a more cell-specific source, allowing for more precise methylation profiling, although leukocyte heterogeneity persists. Although studies investigating histone modification remain limited, emerging evidence suggests that they may play a regulatory role in inflammatory responses and GC signaling.

In summary, future diagnosis and classification of SRNS are likely to benefit from the integration of multiple biomarker types—including DNA methylation, miRNAs, histone modifications, and SNPs—provided that assays are standardized, pre-analytical variables are controlled, and findings are externally validated across centers, thereby enabling more accurate and individualized risk prediction. Using multi-omics integration and advanced statistical modeling, the development of predictive models with high sensitivity and specificity can facilitate early identification and personalized therapeutic strategies for SRNS, ultimately contributing to improved long-term clinical outcomes.

## Discussion: Challenges and opportunities in clinical translation

**Figure 7:**
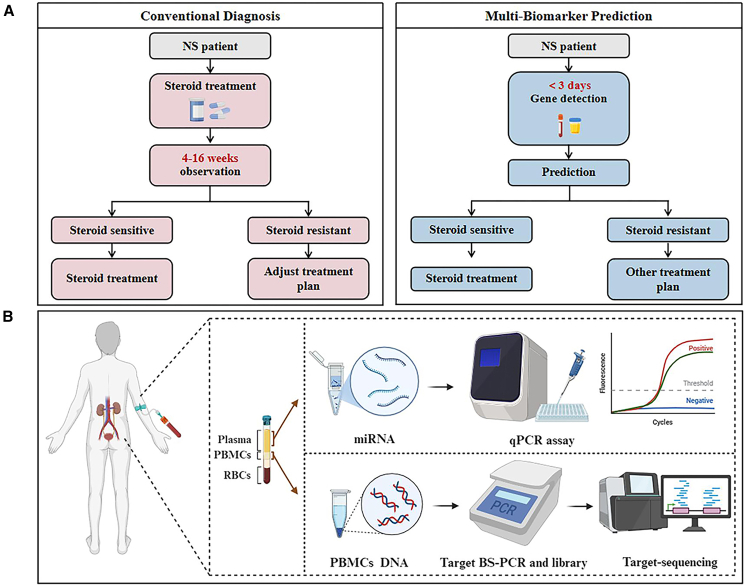
Schematic representation of early SRNS prediction strategies and a multi-biomarker integrated detection workflow (A) Comparison between the conventional diagnostic approach for SRNS and the proposed early prediction model based on multiple biomarkers. This panel illustrates a comparison between the traditional method of assessing steroid responsiveness in NS patients—based on delayed clinical evaluation following GC therapy—and an emerging strategy that incorporates early prediction using a panel of epigenetic biomarkers. (B) Multi-omics workflow for SRNS biomarker profiling. Peripheral blood samples are fractionated into plasma and PBMCs. miRNAs are extracted and quantified via quantitative reverse-transcription polymerase chain reaction (RT-qPCR). Genomic DNA is isolated from PBMCs and subjected to bisulfite conversion, followed by library preparation for targeted bisulfite PCR (BS-PCR) and high-throughput sequencing. This enables identification of epigenetic biomarkers, such as differentially methylated regions (DMRs), associated with SRNS pathogenesis.

Although epigenetic research has made substantial progress in elucidating the molecular basis of SRNS and has identified several promising predictive biomarkers, most clinical studies to date are small, cross-sectional; translation remains challenging and will require standardized assays, prospective validation, and demonstration of clinical utility. Although Whole Exome Sequencing (WES) is utilized by some clinicians to aid in SRNS diagnosis, it fails to identify all cases, and reliance on retrospective GC response assessment can delay diagnosis until progression, compromising the therapeutic window (Figure 7A). Given that the molecular mechanisms underlying GC resistance remain incompletely understood, developing pre-treatment diagnostic strategies and decision thresholds for SRNS is a critical priority. Leveraging experience from oncology and autoimmune diseases and integrating multi-omics genomics, transcriptomics, and epigenomics into SRNS research may accelerate clinical translation toward pre-treatment risk stratification. Accordingly, we propose pre-treatment stratification algorithms that integrate clinical variables with epigenetic biomarkers. We further note that diagnostic timelines, biopsy practices, genetic diagnostic yield, and therapeutic decision-making differ systematically between pediatric and adult SRNS; these differences should be incorporated into risk stratification and trial design to identify high-risk SRNS before prolonged GC exposure.

In recent years, epigenetics has gained considerable attention as a complementary large to genomics for testing and analysis, owing not only to technological advances but also to its intrinsic biological informativeness. At the mechanistic level, three epigenetic modules appear to shape steroid responsiveness in SRNS, namely GR regulation, immune and inflammasome signaling, and podocyte structural programs. Emerging human data suggest coordinated changes across these modules in SRNS; however, the signals remain predominantly correlational and vary by tissue matrix and assay platform, underscoring the need for standardized, multi-cohort validation prior to clinical adoption. Unlike genomics, DNA methylation is a ubiquitous epigenetic modification, and its detection coupled with rigorous data analysis can yield disease-specific insights. Consequently, DNA methylation profiling holds significant promise in SRNS research by providing candidate biomarkers for GC resistance. Furthermore, miRNAs, a class of non-coding RNAs, are increasingly recognized as modulators of GC responsiveness through post-transcriptional regulation. Increasing evidence indicates that miRNAs modulate GR function by targeting GR/*NR3C1* mRNA for degradation or translational repression, thus influencing the sensitivity of patients with SRNS to GC.

Based on existing research, the integrated analysis of DNA methylation, miRNA, and histone modification profiles may facilitate the precise diagnosis of SRNS. Specifically, combining methylation patterns of *SOCS3*, *CASP1*, *NLRP3*, and *NR3C1* with key miRNA and histone profiles could provide a more comprehensive understanding of GC resistance mechanisms, providing critical insights for early diagnosis and accurate subtyping of SRNS (Figure 7B).

Despite its potential, clinical translation faces multiple challenges, including the identification of biomarkers with high sensitivity and specificity, cost, and workflow constraints. Currently, there are no standardized biomarker panels with proven sensitivity, specificity, and clinical feasibility. For miRNAs, many candidates have been reported, but most studies are correlational, lack external validation, and do not fully verify target-pathway mechanisms, limiting robust model development. Histone modification research remains nascent in SRNS, with uncertain functional roles and incomplete regulatory networks.

From a clinical translation standpoint, several practical barriers need to be addressed before epigenetic biomarkers can inform decision-making. Pre-analytical variability across matrices such as PBMCs, whole blood, urine sediment, and cell-free DNA, together with the timing of sampling relative to GC exposure, sample handling, storage, and freeze-thaw cycles, can alter signal magnitude. Analytical sources of variability include platform differences and batch effects and the absence of harmonized protocols and external controls. Clinical utility will require prespecified decision thresholds, reference intervals stratified by age and disease stage, and blinded, multi-center replication with prospective enrollment. Implementation considerations include cost, turnaround time, interoperability with existing laboratory information systems, and alignment with biopsy and genetic-testing pathways. Finally, pediatric and adult workflows differ in diagnostic timelines and treatment algorithms, and study design and reporting should reflect these differences to support generalizability.

Artificial intelligence (AI) has emerged as a powerful tool for managing high-dimensional epigenomic data and supporting evidence synthesis. In SRNS, AI-driven methods show promise for predicting GC responsiveness, integrating multi-omics, and estimating individualized outcomes. AI models are uncovering complex, non-linear associations in large-scale epigenetic datasets, facilitating the selection of biomarker panels with diagnostic utility. AI also enables the integration of multi-omics with clinical parameters to construct models that differentiate SRNS from SSNS and support pre-treatment stratification. These models can support pre-treatment risk stratification and molecular subtyping, improving diagnostic accuracy and informing personalized decisions. Notably, AI tools offer real-time data processing and adaptive learning capabilities, enabling continuous model refinement as datasets evolve. This dynamic adaptability supports precision medicine by facilitating more responsive and individualized clinical decision-making.

Despite the promising potential of AI in epigenetic research, its application in SRNS remains limited by several challenges, including insufficient training datasets and limited model interpretability. To advance clinical translation, future efforts should prioritize interdisciplinary collaboration, the establishment of standardized data frameworks and reporting, and the development of ethical and regulatory guidelines to ensure the safe and effective implementation of AI technologies in the early diagnosis and precision management of SRNS. At the same time, it should be acknowledged that such approaches remain investigational, not ready for standard of care.

Therefore, future research should prioritize functional validation of epigenetic mechanisms and conduct large-scale panel studies to support biomarker discovery. By leveraging AI technologies, statistical modeling can be employed to integrate traditional clinical parameters such as proteinuria and pathological classification with WES and epigenetic biomarkers, enabling the construction of comprehensive and accurate diagnostic models for SRNS. This integrative strategy has the potential to significantly improve the sensitivity and specificity of prediction of GC resistance, facilitate the clinical translation of multi-omics biomarker profiling, and ultimately enhance early detection and long-term results in SRNS patients.

## Limitations of the study

The conclusions of this review are limited by the design and heterogeneity of current human studies linking epigenetic variation to GC responsiveness in SRNS. Available datasets are often small, observational, and enriched for specific ages, histologies, and treatment contexts, which constrains generalizability and precludes causal inference. Reported DNA methylation and miRNA associations are sensitive to tissue matrix and cellular composition and are affected by non-harmonized platforms and analytical pipelines, while evidence for histone modifications remains comparatively sparse. Accordingly, the proposed multi-biomarker risk-stratification framework should be regarded as a conceptual synthesis that requires standardized assays and prospective, pre-treatment multi-center validation before clinical adoption.

## Data and code availability

The data supporting the findings of this study will be made available upon reasonable request to the corresponding author.

## Acknowledgments

This project was supported financially by the Collaborative Innovation Fund of Medicine and Education of 10.13039/501100002703Jiangsu University (JDY2023020), Medical Science and Technology Innovation Project of Xuzhou Municipal Health Commission (XWKYHT20240005), Xuzhou Medical University Affiliated Hospital Science and Technology Development Fund (XYFM202315), and Higher Education Teaching Reform Research Project of 10.13039/501100012217Xuzhou Medical University (XJYLCZX202310).

## Author contributions

Y. Zhu: conceptualization and writing – original draft; Y. Wang: methodology and investigation; C.G.H.: data curation; C.C.: software and validation; O.A.: visualization; Y.F.: resources; H.H.: formal analysis; T.J.: project administration; Y. Zhou: writing – review and editing; X.Z.: supervision; Y. Wu: funding acquisition.

## Declaration of interests

The authors declare no competing interests.
